# Supplementary material for: The interactome of intact mitochondria by cross-linking mass spectrometry provides evidence for coexisting respiratory supercomplexes
Source: Mol Cell Proteomics. 2017 Dec 8;17(2):216–32. doi: 10.1074/mcp.RA117.000470 (PMC5795388; doi:10.1074/mcp.RA117.000470)
Supplement: Supplemental Data [file supp_RA117.000470_133922_0_supp_23978_fzffwf.zip › spectra_annotation/mito_spectra_annotation/355-1-12-1-82-1.pdf]

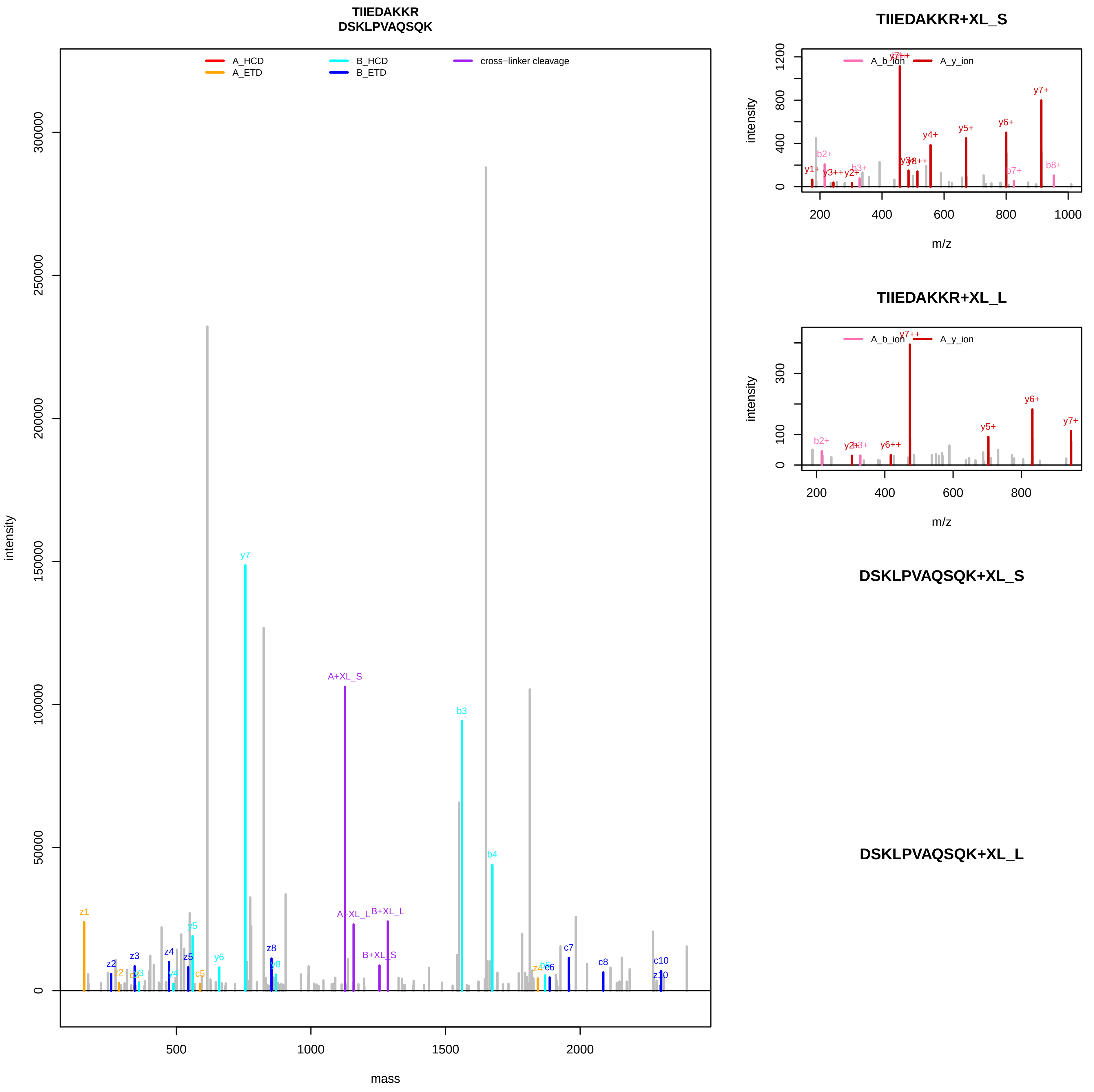
TIIEDAKKR
DSKLPVAQSQK
A_HCD
A_ETD
B_HCD
B_ETD
cross−linker cleavage
intensity
mass
A+XL_S
A+XL_L
B+XL_L
B+XL_S
y7
b3
b4
y5
y6
y8
z1
z2
z3
z4
z5
z8
c5
c6
c7
c8
c10
z10
b6
0
50000
100000
150000
200000
250000
300000
500
1000
1500
2000
TIIEDAKKR+XL_S
A_b_ion
A_y_ion
m/z
y1+
b2+
y3++
y2+
b3+
y7++
y3+
y8++
y4+
y5+
y6+
y7+
b7+
b8+
TIIEDAKKR+XL_L
y6++
DSKLPVAQSQK+XL_S
DSKLPVAQSQK+XL_L
